# Gene-Specific Targeting of DNA Methylation in the Mammalian Genome

**DOI:** 10.3390/cancers11101515

**Published:** 2019-10-09

**Authors:** Arthur Urbano, Jim Smith, Robert J. Weeks, Aniruddha Chatterjee

**Affiliations:** 1 Department of Pathology, Dunedin School of Medicine, University of Otago, 56 Hanover Street, Dunedin 9054, New Zealand; urbar374@student.otago.ac.nz (A.U.); jimasmith1@gmail.com (J.S.); 2 Maurice Wilkins Centre for Molecular Biodiscovery, 3A Symonds Street, Private Bag 92019, Auckland 1142, New Zealand

**Keywords:** DNA methylation, cancer, CRISPR, editing, epigenetics

## Abstract

DNA methylation is the most widely-studied epigenetic modification, playing a critical role in the regulation of gene expression. Dysregulation of DNA methylation is implicated in the pathogenesis of numerous diseases. For example, aberrant DNA methylation in promoter regions of tumor-suppressor genes has been strongly associated with the development and progression of many different tumors. Accordingly, technologies designed to manipulate DNA methylation at specific genomic loci are very important, especially in the context of cancer therapy. Traditionally, epigenomic editing technologies have centered around zinc finger proteins (ZFP)- and transcription activator-like effector protein (TALE)-based targeting. More recently, however, the emergence of clustered regulatory interspaced short palindromic repeats (CRISPR)-deactivated Cas9 (dCas9)-based editing systems have shown to be a more specific and efficient method for the targeted manipulation of DNA methylation. Here, we describe the regulation of the DNA methylome, its significance in cancer and the current state of locus-specific editing technologies for altering DNA methylation.

## 1. Introduction

Cancer is one of the leading causes of mortality worldwide and constitutes a major public health burden, despite the continued emergence of novel therapeutic approaches and improved clinical management [1]. Metastasis, or the ability of tumor cells to spread to distant organs in the body, is one major hallmark of neoplastic progression and is responsible for 90% of cancer-related deaths [2,3]. Over the past two decades, many studies have shown that epigenetic changes are closely associated with each of the steps involved in tumor development and progression [4].

Much of the existing evidence regarding epigenetic aberrations in cancer are based on the initial events of tumorigenesis, whilst less is known about the epigenetic events that can lead to metastasis [5]. Primary tumor cells require additional changes for successful metastasis, as even though these tumor cells have acquired cancer-specific mutations, less than 0.01% of cells that enter the circulation are able to metastasize [3]. This notion is supported by extensive sequencing data which indicates that genetic mutations alone are insufficient for successful metastasis [6]. Epigenetic changes are now postulated as having an important role in primary cancer cell progression, contributing to the acquisition of additional properties that are essential for cancer metastasis [2,7,8,9].

DNA methylation, alongside histone modifications and non-coding RNAs, is one of the major mechanisms of epigenetic regulation and has a well-established role in the pathogenesis of many diseases, including cancers [6]. Aberrant methylation was first reported in human cancers in 1983 [10]. However, little is known about the mechanisms underlying methylation changes during cellular differentiation and tumorigenesis. Moreover, establishing whether a causal relationship exists between DNA methylation and transcription has been difficult thus far. Fortunately, the recent development of targeted tools for manipulating DNA methylation offers the opportunity to address these gaps in our understanding.

Here, we describe the regulation of DNA methylation in the mammalian genome and explore the current state of DNA methylation-editing technologies. Further, we provide details of the published work in this field thus far, the targeted editing systems currently available, and finally, the potential implications of successful methylation-editing in cancer therapy development.

## 2. Regulation and Maintenance Mechanisms of DNA Methylation

DNA methylation is involved in many different regulatory activities throughout the genome, including the regulation of gene expression, genomic imprinting, X inactivation, and maintenance of genomic stability, as well as silencing of retroviral elements [11]. More than half of genes contain cytosine-guanine dinucleotide (CpG)-rich regions called CpG islands, commonly found within the promoter regions of important regulatory genes [12]. Cytosine DNA methylation at promoters or distal regulatory elements is generally associated with transcriptional repression or gene silencing via local chromatin conformational change, prevention of transcription factor binding, or via binding of the methyl-CpG-binding domain proteins. Conversely, CpG islands found within the promoters of housekeeping and tumor-suppressor genes are commonly hypomethylated to activate gene transcription. Eumethylation of these hypomethylated promoters facilitates optimal chromatin conformation and the recruitment of regulatory proteins which are required for transcription to occur [11,13,14].

**Figure 1:**
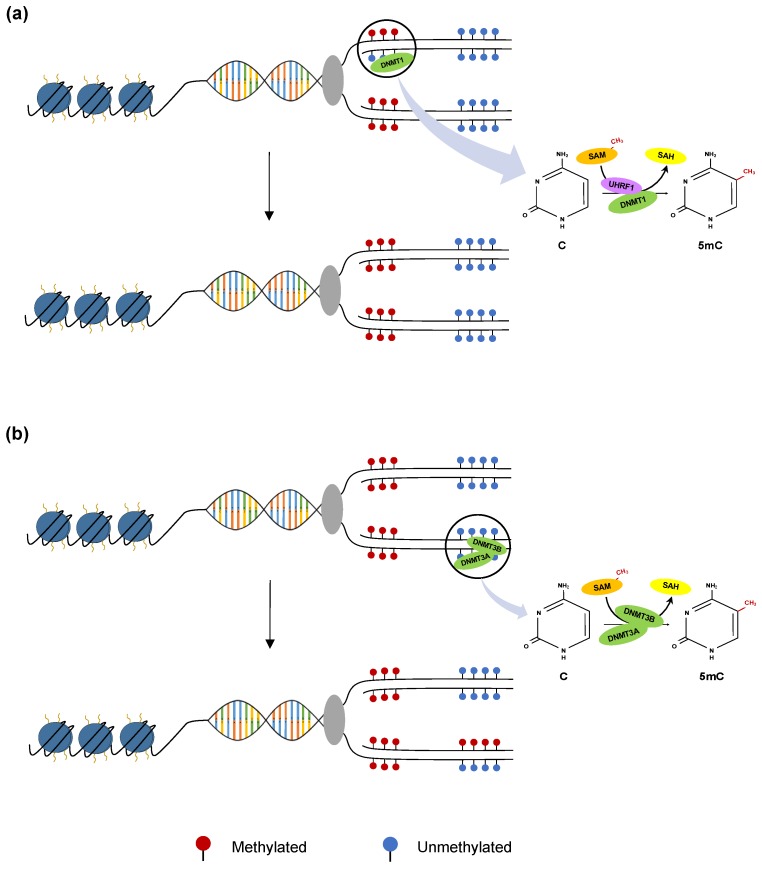
Regulation of the DNA methylome by methyltransferase enzymes (**a**) Maintenance of methylation. Shown is the action of DNA methyltransferase (DNMT)1 (green) at the replication fork catalyzing the methylation of hemimethylated DNA during replication. Ubiquitin-like containing PHD and RING finger domains 1 (UHRF1) (purple) acts as an important co-factor in the recruitment of DNMT1. S-adenyl methionine (SAM) acts as a donor of the required methyl group and is converted to S-adenosyl homocysteine (SAH) during the addition of methylation (CH3) marks (red) (**b**) De novo methylation. Shown are the replication-independent de novo methyltransferases, DNMT3A and DNMT3B, catalyzing the addition of new methylation marks at previously unmethylated cytosine-guanine dinucleotide (CpG) sites. SAM is shown as the methyl group donor for the conversion of cytosine to 5-methylcytosine (5mC).

In mammals, DNA methylation occurs almost exclusively within the context of CpG dinucleotides, via covalent addition of a methyl group to the fifth carbon position of cytosine residues. This process is a post-replication chemical modification catalysed by the DNA methyltransferase (DNMT) family of enzymes. DNMTs transfer a methyl group from the donor molecule S-adenyl methionine (SAM) to cytosine, producing a 5-methylcytosine (5mC) residue (Figure 1) [14].

### 2.1. Methylation by De Novo and Maintenance Mechanisms

DNMT3A and DNMT3B are the two DNA methyltransferase enzymes which catalyze de novo DNA methylation. De novo methylation is essential for establishing DNA methylation patterns during embryonic cell differentiation and germ cell line specification during development [15]. DNMT3A is especially required for the establishment of methylation of imprinted genes in germ cells [16,17], whilst DNMT3B is responsible for the methylation of pericentromeric satellite regions [18,19].

Both enzymes act independently of replication and show equal preference for both unmethylated and hemimethylated DNA (Figure 1) [18]. DNMT1 is responsible for maintaining DNA methylation in a replication-dependent manner. It ensures fidelity of established epigenetic patterns after DNA synthesis [13]. This interaction is supported by a larger complex of chromatin-associated enzymes that allow for precise control of global methylation inheritance [20].

### 2.2. Active and Passive DNA Demethylation

**Figure 2:**
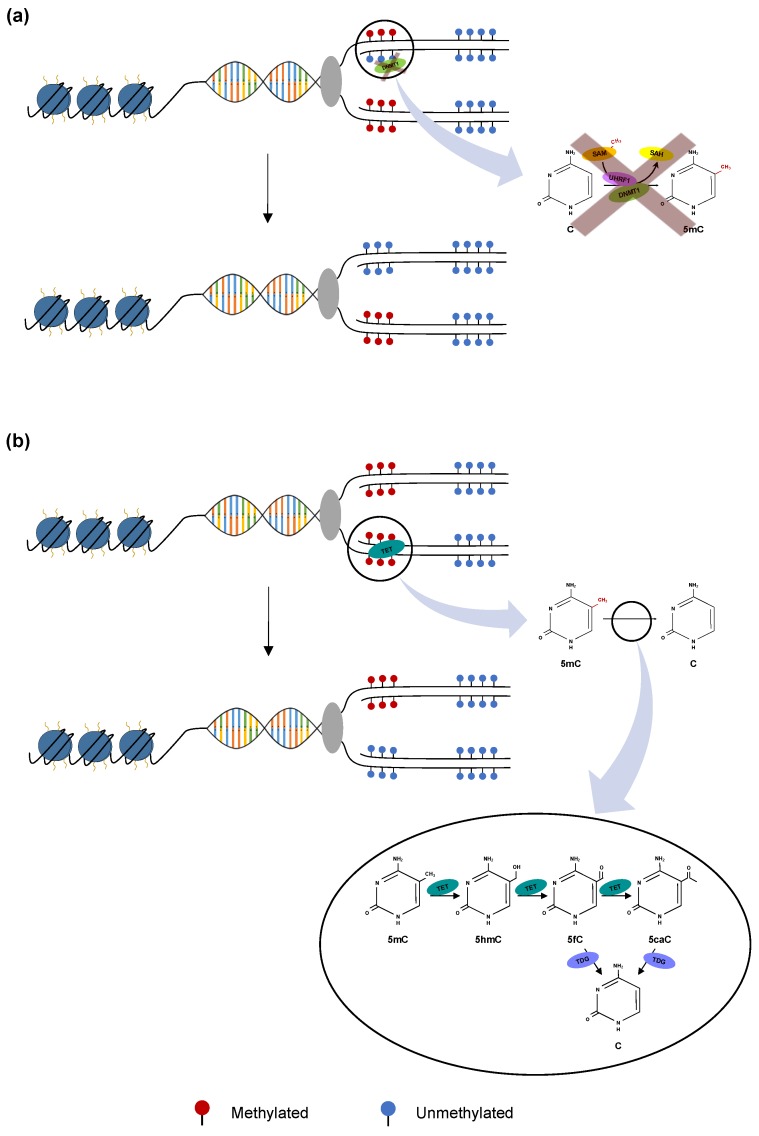
Mechanisms of DNA demethylation (**a**) Passive demethylation. This process occurs during replication wherein one or more limiting factor (i.e., compromised DNMT function, absence of SAM) prevents methylation maintenance and results in the subsequent loss of 5mC residues. (**b**) Active demethylation. Shown are the ten-eleven translocation (TET) enzymes (TET1, TET2 or TET3) (teal) catalyzing stepwise oxidation of 5mC. 5mC is first converted to 5-hydoxymethylcytosine (5hmC) which is further oxidized to 5-formylcytosine (5fC) and finally to 5-carbocylcytosine (5caC). 5fC and 5caC intermediates can be recognized and removed by thymine DNA glycosylase (TDG) (violet). They are then replaced with an unmethylated cytosine nucleotide to complete the base excision repair (BER) process.

Alternatively, loss of DNA methylation, or demethylation, can occur through either passive or active pathways. Passive demethylation occurs when re-methylation is inhibited during DNA replication leading to loss of 5mC residues, such as when the DNMT function is compromised or essential cofactors like SAM are absent (Figure 2a) [21]. In contrast, active demethylation is replication-independent and involves the conversion of 5mC residues to unmethylated cytosine, via either enzymatic oxidation or deamination followed by base excision repair (BER) (Figure 2b) [22,23].

The ten-eleven translocation (TET) family of dioxygenase enzymes (TET1, TET2, and TET3) mediates the initial step of DNA demethylation oxidizing 5mC to 5-hydroxymethylcytosine (5hmC). The deamination pathway operates in the same manner wherein 5mC and 5hmC residues are enzymatically converted by the activation-induced deaminase (AID) and apolipoprotein B mRNA-editing enzyme complex (APOBEC) family into thymine and 5-hydroxymethyluracil (5hmU), respectively. TET-mediated demethylation is more biologically relevant in mammals than the AID/APOBEC pathway; Nabel et al. have shown that AID/APOBEC-induced deamination occurs with lower efficiency, due to an increased affinity for unmodified cytosine as compared to 5mC [24].

## 3. DNA Methylation in Cancer

Aberrant DNA methylation changes have been implicated in a number of pathological conditions, including cancer. In comparison to normal somatic tissues, the cancer methylome is typically characterized by a pattern of global hypomethylation coupled with site-specific promoter hypermethylation [25,26,27,28]. This genome-wide hypomethylation is associated with chromosomal instability, loss of genomic imprinting and the reactivation of transposable elements, each of which contributes to the aberrant gene expression patterns observed during tumor development and progression [26,27,28,29]. Dense hypermethylation of promoter CpG islands is an important mechanism for regulating transcriptional activity under normal physiological conditions. This hypermethylation prevents inappropriate transcriptional activation by blocking the access of transcriptional machinery to the gene promoter. In tumorigenesis, however, aberrant hypermethylation is commonly observed within the promoters of key regulatory genes involved in preventing neoplastic change, including cell cycle processes, DNA repair, and apoptotic pathways.

### 3.1. DNA Methylation as a Driving Force for the Functional Hallmarks of Cancer

Hanahan and Weinberg [30] have identified six physiological and molecular “hallmarks of cancer”. They propose that the majority of cancers aim to acquire the same set of “hallmark” functional capabilities throughout their development and progression. These capabilities are: (1) self-sufficiency in growth signals; (2) evading apoptosis; (3) insensitivity to antigrowth signals; (4) tissue invasion and metastasis; (5) sustained angiogenesis; and (6) limitless replicative potential. A number of genes associated with tumor development and progression are silenced by aberrant DNA methylation; several examples of this are detailed in Table 1.

Moreover, two further hallmarks have since been described: Deregulated metabolism and immune evasion [37]. Immune evasion is an emerging hallmark with a strong epigenetic component, wherein epigenetic mechanisms are employed by cancer cells to modify and dampen the normal immune response, enhancing tumor survival [38]. For example, in ovarian tumors, immunoprotective genes were shown to be epigenetically silenced, resulting in the repression of T helper 1-type chemokine production [39]. This hallmark is also an emerging target for immunotherapy in combination with epigenetic therapy. Drugs blocking DNMT activity have led to remarkable improvement in patient responses [38,40,41,42], indicating that epigenetic modulation is important for avoiding immune destruction in cancers.

Epigenetic aberrations were also proposed as a hallmark of cancer [43,44,45], however, it was argued that epigenetics is a molecular mechanism contributing to the acquisition of these characteristics and not a defined property of cancer. The disruption of epigenetic mechanisms allows tumor cells to gain hallmark properties in the same manner as genetic mutations. One convincing piece of evidence supporting this concept is the observation that promoter hypermethylation leads to loss of function of several key genes.

DNA methylation can be reversible and can lead to the activation of gene transcription. Importantly, this can occur in potential oncogenes. The first evidence of hypomethylation associated with high level of expression was reported for the *BCL2* gene in lymphocytic lymphoma [46]. This was followed by reports showing the same phenomenon in proto-oncogenes such as *RRAS* in gastric cancers [47], and *MAGE* family genes and *GPR17* in lung and head and neck cancers [48]. Normally silenced by methylation, demethylation of the *HIF-1α* promoter enables HIF-1α protein to bind to its own promoter, auto-transactivating gene expression, and resulting in a hypoxic response [49]. Overexpression of HIF-1α has critical implications in energy metabolism, angiogenesis, cell survival, and tumor invasion, all which are important for cancer growth [50]. More recent work reports that hypoxia-induced loss of TET family of enzymes resulted in the hypermethylation of various gene promoters, conferring a selective advantage for tumor cells [51].

Notwithstanding the substantial body of evidence correlating high levels of promoter methylation with transcriptional silencing, an increasing number of examples now identify contexts in which this observation does not appear to hold true. In line with the dynamism of DNA methylation, an increasing number of published articles identify that high levels of promoter methylation also appear to correlate with active gene transcription in some contexts. This phenomenon has been demonstrated for *EBF3* [8], *MGMT*, *HOXD12*, and *GATA4* [52] genes in melanoma, *WT1* in acute myeloid leukaemia [53], *TIMP2* in cervical cancer [54], and *hTERT* in multiple cancer cell lines [55,56,57,58,59]. These examples suggest that in specific contexts, high levels of DNA methylation may in fact facilitate an increase in transcriptional activity, which challenges the current dogma of promoter DNA methylation as a solely transcriptional silencing mechanism.

### 3.2. Establishing Causality between DNA Methylation and Transcriptional Control

Thus far, it has not been possible to conclusively establish causality between promoter methylation and subsequent expression change with the current drugs available for manipulating DNA methylation. DNA methyltransferase inhibitors (DNMTi) are the mainstay drugs for therapies, mainly used in the treatment of myelodysplastic syndrome and acute myeloid leukemia [60,61]. DNMTi such as 5-azacytidine treatment inhibits replication by incorporating into the groove of DNMTs and preventing the generation of 5mC residues [62]. However, DNMTi is a global methylation modifier and so cannot demonstrate the direct causal relationship between methylation status at a specific locus and the corresponding transcriptional regulation. DNMTi have been used experimentally in the treatment of cell lines. Many examples have shown the removal of promoter methylation after treatment with 5-azacytidine or decitabine. In genes with previously dense methylation, increased expression was observed following the removal of methylation marks. In theory, every locus is demethylated evenly, however, it was demonstrated that 5-azacytidine does not demethylate every part of the genome in the same fashion. These results show that even with the success of the decitabine treatment, it is still a global demethylation process. The question remains as to what level or extent promoter methylation is involved in this expression change with regards to causality. Elucidating the nature of this relationship will therefore only be possible with the advent of new gene-specific targeting tools.

## 4. Gene-Specific Editing of DNA Methylation in the Mammalian Genome

As we have seen, DNA methylation and demethylation play a critical role in regulating gene expression across a vast range of physiological and pathological contexts and technologies for manipulating DNA methylation at a specific region are crucial for understanding this regulation. However, the development of such technologies has proven to be very difficult. Previous epigenetic technologies like zinc finger proteins (ZNF) and transcription activator-like effector proteins (TALEs) have been utilized. ZNFs and TALEs are modular DNA-binding proteins, whose DNA-binding domains (DBD) are engineered to recognize specific target nucleotides sequences [63,64].

### 4.1. ZNFs and TALEs

The first DNA-binding proteins to be utilized in targeted editing were the eukaryotic ZNFs, and represented the beginning of a new era in genomic and epigenomic manipulation [65]. ZNF are transcription factors, comprising protein motifs or fingers that recognize and bind three DNA nucleotides. Different ZNF modules are used in combination, based on their respective affinities for a particular three base sequence, to target specific genomic regions. ZNF DNA binding domains are therefore commonly fused with a nuclease or other effector protein, to mediate a site-specific genetic or epigenetic response [63,65,66,67].

TALEs, isolated from the *Xanthomonas* bacteria, were next developed for targeted editing [65]. TALEs are dimeric transcription factors or nucleases, assembled from arrays of amino acid modules. Like ZNF proteins, TALEs allow for customizable, sequence-specific DNA binding. However, TALEs have the ability to bind individual bases at a target locus. Similarly to ZNF-based tools, TALEs fused with specific effector proteins have the capacity to induce a particular effector response at a select target locus [65,68].

Though ZNF- and TALE-based technologies provided a platform for genomic and epigenomic editing at a single-locus, these techniques are difficult and laborious, with each targeting site requiring a complete re-design and re-engineering of a new set of proteins. In comparison, the emergence of clustered regulatory interspaced short palindromic repeats (CRISPR)-based technologies provides much simpler and easily-targetable systems and provides an equal or greater level of editing efficacy than these existing options [65].

### 4.2. CRISPR-Based Editing Systems

**Figure 3:**
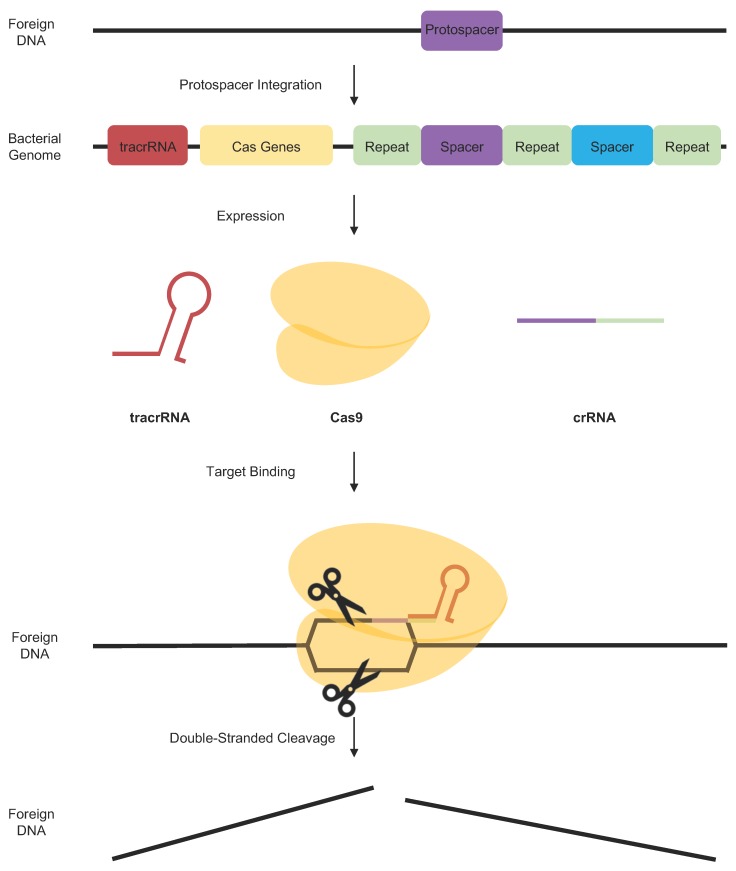
Overview of Type II *S. pyogenes* clustered regulatory interspaced short palindromic repeats (CRISPR)-Cas System. Shown is a schematic overview of the type II CRISPR system utilized by *S. pyogenes*. Foreign protospacer DNA from foreign exogenous elements is acquired by Cas9 and integrated into the CRISPR loci as a spacer. The CRISPR system recognizes the same foreign agent when it invades the cell again. This allows the transcription and expression of the corresponding trans-activating CRISPR RNA (tracrRNA) and CRISPR RNA (crRNA) along with the Cas9 nuclease. These complex binds to the invading element, guided by tracrRNA and crRNA, and induces double-stranded cleavage of the foreign DNA as an adaptive immune response.

The CRISPR-Cas system was first discovered as an adaptive immune response mechanism of bacteria against invading viruses. The CRISPR loci are composed of a clustered set of *Cas* genes that are flanked by identical repeat nucleotide sequences with “spacers” sitting in between them. These nucleotide spacers were acquired by Cas enzymes from exogenous protospacers following the invasion of viruses. In the event of re-invasion by the same virus, the spacers recognize and target the same specific genetic element for cleavage with the Cas9 endonuclease enzyme (Figure 3) [69].

#### 4.2.1. Basic Components of CRISPR

The type II CRISPR system utilized by *Streptococcus pyogenes* (Figure 3) is the best characterized system for genome and epigenome editing, consisting of the Cas9 nuclease, a CRISPR RNA (crRNA), and trans-activating CRISPR RNA (tracrRNA). The crRNA hybridizes with the tracrRNA, recruits Cas9 and binds to foreign protospacer elements [70]. To simplify the application of this system, the two RNAs can be fused together forming a chimeric, single-guide RNA (sgRNA) [71]. Cas9 can be directed to almost any target through modification of this guide RNA (gRNA) molecule by alteration of the 20 bp guide sequence in the spacer. In the CRISPR-Cas9 system derived from *S. pyogenes*, the target sequence should immediately follow a 5′-NGG protospacer-adjacent motif (PAM), although different Cas9 orthologs from other bacterial species have different PAM requirements. PAM recognition is required for ATP-independent strand separation, and for gRNA complexing with target genetic elements [71].

For epigenome editing, cleavage of the DNA sequence is not required. As such, the Cas9 nuclease is deactivated to remove its catalytic activity [70]. Nuclease-deficient Cas9 is prepared by single-amino-acid substitutions of Asp^10^ to Ala^10^ and His^840^ to Ala^840^ in the HNH and RuvC-like domains of Cas9 [69]. Currently, research is ongoing to optimize and develop the use of CRISPR-deactivated Cas9 (dCas9) for targeted editing of DNA methylation.

**Figure 4:**
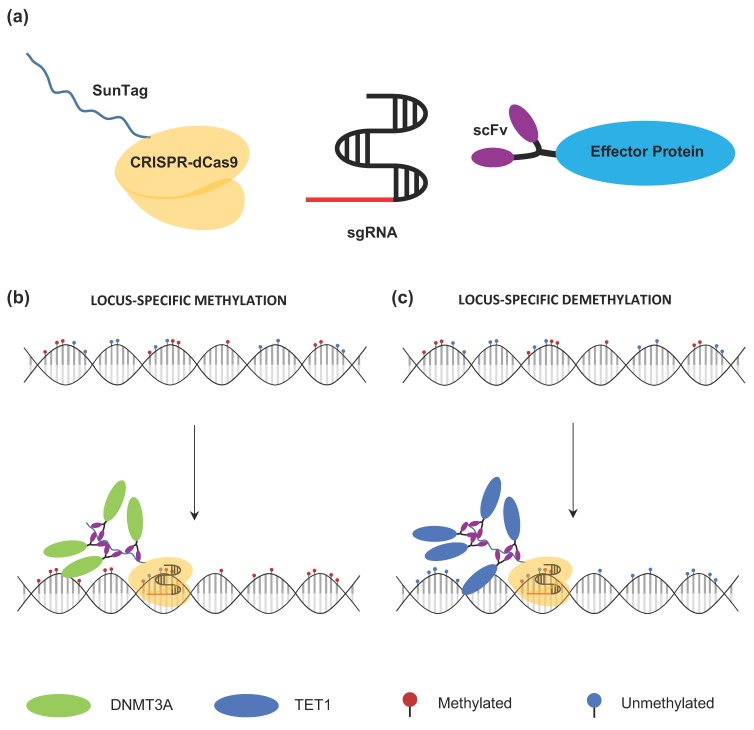
Components of the CRISPR-deactivated Cas9 (dCas9)-based methylation-editing system. (**a**) Depicted are three basic components required for selective methylation or demethylation of a target locus: The CRISPR-dCas9 protein (yellow, top left); a unique gRNA sequence (red) fused to the single-guide RNA (sgRNA) plasmid construct; and the effector protein domain (DNMT3A for methylation or TET for demethylation). (**b**) Locus-specific methylation strategy. The major difference for methylation and demethylation for specific locus editing is the epieffector used in the system. For methylation, the epieffector DNMT3A (green) catalyzes the addition of methyl marks. (**c**) Locus-specific demethylation strategy. TET1 enzyme (blue) is used as an epieffector for the removal of methyl marks.

The basic requirement for CRISPR epigenome editing consists of three essential parts: A DNA-binding targeting protein, an effector protein and a unique gRNA sequence (Figure 4a). The CRISPR-dCas9 system is an ideal targeting protein complex, due to its ability to be targeted by guide RNAs to multiple sites and its insensitivity to CpG methylation [72,73]. Fusion of the effector protein component to the CRISPR-dCas9 targeting protein represents the first CRISPR-based tool capable of modulating DNA methylation at a target locus [65]. For example, the DNMT3A and TET dioxygenase enzymes have been fused to dCas9 for targeted epigenome editing methylation and demethylation, respectively (Figure 4b,c).

#### 4.2.2. Strategies for CRISPR-dCas9-Based Targeted Methylation

Deactivated Cas9 functions as a DNA binding domain [74]. For methylation, the dCas9 is fused with the catalytic domain of DNMT3A (Figure 4b). DNMT3A, as previously discussed, is required for de novo methylation, preferentially methylating CpG sites [75]. Moreover, its catalytic domain alone shows enzymatic activity in transfected cells [76].

CRISPR-dCas9-DNMT3A stimulates de novo deposition of DNA methylation at a specific site, often with the goal of inducing transcriptional repression. However, improvisation and optimization of the system has been performed to overcome technological challenges and to improve specificity, efficiency, delivery, and cytotoxicity.

One of these improvements to the CRISPR system was the utilization of CRISPR-based hybrid proteins. One study utilized the direct fusion of dCas9 to the catalytic domain of DNMT3A (dCas9-DNMT3ACD) through a flexible Gly4Ser linker. This construct induced an increase of 60% in CpG methylation at the *BACH2* loci in human embryonic kidney cells (HEK293T) [77]. To increase the percentage of methylation, chimeric MTase fusion proteins were developed. A study by Stepper et al. [78] using a DNMT3A-DNMT3L chimeric fusion protein showed a greater percentage of induced methylation as compared to dCas9-DNMT3ACD. Additionally, a chimeric MTase of three dCas9 fused to DNMT3A, DNMT3L, and Krupple-associated box (KRAB) proteins, respectively, demonstrated further improvement in methylation efficacy [79]. Previous reports have shown that DNMT3L has the ability to enhance de novo methylation by forming hetero-tetramers with the catalytic domain of DNMT3A [80,81]. Therefore, multimerization of MTases was developed to enhance the activity for long-range methylation editing. An example of this is the SUperNova TAGging (SunTag) system developed by Tanenbaum et al. [82]. SunTag refers to a repeating peptide array with the capacity to recruit multiple copies of an antibody-fusion protein at a target locus. By adopting this strategy, Huang et al. [83] developed a dCas9-SunTag-DNMT3A system that was able to recruit multiple copies of DNMT3A to the *HOXA* genomic locus.

Each of the aforementioned CRISPR-dCas9 systems have a relatively long duration of application, ranging from three to thirty days. Hence, Lei et al. developed a dCas9-MQ1 fusion protein (or M.SssI), derived from *Mollicutes spiroplasma*, to achieve more rapid targeted methylation within seventy-two hours [84]. In this study, a direct mouse zygote injection strategy was utilized to target de novo methylation on the imprinted *Igf2/H19* region. The rapid editing response achieved with this system makes this tool potentially applicable during early embryogenesis. However, high levels of off-target effects were reported with this system. Furthermore, to improve targeting, the MTase was split into two parts, the N-terminal and C-terminal domain, with the latter fused to dCas9 to guide the complex of methyltransferase to targeted CpGs. Overall, many strategies based on the CRISPR system for methylation targeting utilizing different DNA binding platforms and methyltransferases have been devised and continue to be optimized. These are detailed below in Table 2.

#### 4.2.3. Strategies for CRISPR-dCas9-Based Targeted Demethylation

The TET hydroxylase catalytic domain fused to dCas9 is currently the main strategy for demethylation of 5mC marks (Figure 4c). Before the advent of CRISPR, both ZNFs and TALEs were used as binding platforms for TET enzymes and both systems were able to induce transcription at targeted loci [89,90]. However, for the same reasons of cost and difficulty discussed earlier, their applications are limited. Since 2016, several demethylation studies have been published using CRISPR-dCas9 systems (Table 3). Generally, each system utilizes the CRISPR-dCas9-TET1 fusion protein paired with a programmable 20 nucleotide sgRNA guide homologous to the target locus. The first study using a transient and lentiviral-based dCas9-TET1 system showed selective targeting of the *BRCA1* promoter to induce robust gene expression [91]. Xu et al. employed a strategy of modifying sgRNA by inserting bacteriophage MS2 RNA elements into the conventional sgRNA, allowing for direct tethering of MS2-fused Tet1CD proteins [92]. Another successful strategy utilized the SunTag multimerization system which was further improved by Morita et al. to enhance TET1 recruitment and demethylation. The authors changed the length of the SunTag linker from five to twenty-two amino acids, allowing more efficient recruitment of multiple copies of antibody-fused TET1 and achieved up to 90% demethylation both in vitro (different cell types) and in vivo (mouse embryonic model) [84]. With this dCas9-TET1 fusion system, multiple studies have demonstrated demethylation with an associated increase of mRNA expression in target genes [85,92,93].

Recently, several studies have been published applying this fusion protein demethylation system to target other elements outside of gene promoters, giving insight into the applicability of this system in other contexts. For example, it was applied to demethylate a distal enhancer (*MyoD*), promoting myogenic reprogramming in fibroblasts [85]. Moreover, it was able to demethylate CGG repeats in Fragile X syndrome-induced pluripotent stem cells [94], and to reactivate the silenced *FMR1* by activating its promoter, which induced sustainable reactivation in a human-mouse chimeric model [94]. These achievements demonstrate some of the possible applications of this system in analyzing the causality of disease-associated DNA methylation aberrations and for future therapeutic applications. This system, however, requires further optimization and research to be fully established in in vivo experiments.

A new dCas9 system without TET activity, the dCas9-R2 system, has also recently been developed [96]. With this system, DNMT1 is recruited by an R2 loop, thus inhibiting DNMT1 enzyme activity at the specific target site and preventing DNA methylation maintenance during replication. This system shows similar efficiency to the current dCas9-TET1 system, has better targeting accuracy (the editing window is within approximately 100 bp of the target site) and avoids the potential side effects of exogenous TET protein expression.

Another potential demethylation strategy is the use of DNA glycosylase enzymes instead of TET enzymes. For example, thymidine DNA glycosylase (TDG) is an enzyme involved in methylcytosine demethylation (as discussed previously). An earlier study by Gregory et al. showed that targeted DNA demethylation using TDG can upregulate gene expression [97]. A recent study using *Arabidopsis* ROS1 5mC DNA glycosylase (ROS1CD) demonstrated a decrease in methylation of targeted promoters followed by increased transcription. This ROS1CD glycosylase directly excises 5mC and initiates substitution for unmodified cytosine, however, further optimization is required before widespread adoption of these glycosylase strategies is possible [95].

## 5. Application

### 5.1. In Vivo Applications

Following the success of the in vitro experiments, the next step for these epigenomic engineering techniques is applying them in vivo and exploring the potential for therapeutic applications. Most studies in cancer that have utilized the dCas9 editing system have been performed in vitro, with only three studies to date having applied these technologies to in vivo situations [84,93,98]. Morita et al. achieved up to 90% demethylation of a target loci within an embryonic mouse model [93]. In a separate study, mouse primary T-cells were utilized to stabilize *Forkhead box P3* (*Foxp3*) using the dCas9 system [98]. Another study using a dCas9-MQ1 fusion protein utilized in vivo zygotic targeting in mice via microinjection [84]. The fact that only a few in vivo studies have been performed to date utilizing this engineering tool in vivo indicates that this system is still relatively new and further optimization is required. In comparison, many active Cas9-based systems have succeeded in in vivo genomic editing across multiple tissue types, including muscle, liver, and brain, to either produce desired mutations or correct mutations causing diseases [99,100,101,102].

### 5.2. Potential Therapeutic Applications

Drugs for the modulation of DNA methylation have shown preclinical promise for slowing tumor progression [103]. Moreover, in addition to cancer therapies, these drugs have been trialed in the management of neurodegenerative diseases including Alzheimer’s, Parkinson’s, and Huntington’s diseases [104]. Unfortunately, small molecule inhibitors such as decitabine act via broadly inhibiting the enzymatic activity of epigenetic effectors, and consequently, doses are frequently limited by toxicity after administration. We propose that control over gene regulation via epigenetic modulation will become an increasingly valuable tool with potential for novel therapeutic application.

Of new interest in the realm of immunotherapy is the activation of endogenously methylated sequences (e.g., cancer-testis antigens) which are normally suppressed in somatic cells. Activation of these genes can give rise to neoantigens in treated cells, increasing the immunosurveillance capability of the host. The activation of these genes generates a state of viral mimicry, wherein the treated cancer cells misinterpret this activation as being due to infection by an exogenous virus and mount an immune response [105]. The prospect of utilizing locus-specific methylation-editing technologies in a therapeutic setting is an exciting one [106]. However, further work to improve these methylation-editing tools and to characterize the immune responses to engineered epigenome editing proteins is required before they can be applied clinically.

## 6. Technical Considerations

### 6.1. Off-Target Effects of CRISPR-dCas9 Tools

Despite the use of a unique, programmable 20 nucleotide gRNA sequence, CRISPR-dCas9-enzyme fusion proteins show variable levels of off-target localization, which has been reported from ‘ChIP-seq’ analysis of genome-wide DNA mapping [107,108,109,110]. These off-targets effects are attributed to the presence of the 5–7 bp protospacer sequence preceding the PAM sequence [108,109,110]. Off-target effects are thought to occur either because of mis-recognition by the dCas9-sgRNA complex and subsequent binding at an alternative locus, or via accidental methylation by the DNA methyltransferase component at non-specific loci. The functional consequences of these off-targets are, however, not clear as they do not necessarily result in gene transcription or chromatin accessibility changes as demonstrated by RNA-sequencing (‘RNA-seq’) and DNase I hypersensitivity sequencing [72,107]. Whether or not there is a clear biological consequence of these off-target effects, there are legitimate concerns when attempting to perform site-specific manipulation of the genome. In vertebrates, around 60–80% of CpG sites are highly methylated and only a small fraction are unmethylated or partially methylated [111], making it hard to determine the global effects of methylation. The dynamic state of the methylome also poses a greater challenge. In contrast to the sequence of DNA, DNA methylation is variable and can be modified during cell proliferation and differentiation. Off-target assessments should then be included in future studies to validate the efficacy of each targeting experiment.

### 6.2. Controls

Use of controls is always important in any experiment. There is a possibility that DNMT and TET catalytic domains have the ability to independently induce methylation modifications without the dCas9-mediated targeted deposition. Thus, it is important to validate any observed changes in DNA methylation or gene expression by including multiple controls, such as with catalytically inactive TET1 or DNMT and with scrambled gRNAs or without gRNAs. This ensures that the intended epigenetic modification is directly responsible for generating the observed phenotype.

### 6.3. Techniques Used for Further Analysis Post-Methylation-Editing

Studies to date have shown that the degree of DNA methylation or demethylation induced by dCas9-based tools is not always proportional to the corresponding gene repression or activation effect [85,112]. However, it is difficult to ascertain at this point whether this is a feature of the tools used, of the gene itself, or of the methods used to assess gene expression. Accordingly, this highlights the significance of utilizing more specific and accurate assays to assess and confirm targeted DNA methylation or demethylation and verify specificity. Chromatin immunoprecipitation (ChIP)-qPCR can be used to check the recruitment of dCas9 to a genomic target locus. Additionally, evaluating the methylation status of target loci can be performed using targeted bisulfite sequencing techniques and the assessment of expression changes associated with targeted modification of methylation can subsequently be performed using quantitative reverse transcription PCR (RT-qPCR) or other gene expression analysis methods [113]. Moreover, if the methylation modification is known to show a change in cellular behavior or identity, cell-type-specific assays should be performed [114]. Lastly, single-molecule real-time (SMRT) sequencing techniques can be used to differentiate modified nucleotides including 5hmC, 5mC, and 6mA in modified cells [115].

### 6.4. Expression of the dCas9 Effector and gRNA

Most experiments for epigenome editing are conducted on cell lines such as HEK293 or HeLa after transient transfection of DNA vectors. However, for robust epigenetic modifications, stable transduction and long-term expression of the dCas9 complex is likely to be necessary, especially when applying these systems to primary cells or pluripotent cells [93,116,117]. Stable cell lines expressing fusion proteins have been shown to be more effective compared to standard transfection methods. dCas9 proteins are large and dCas9 fusion proteins are even larger, thus making them difficult to deliver to cells. Viral methods and cationic lipid delivery are common methods for efficient delivery of dCas9 coding sequences [118,119]. The expression of transduced cells can be identified by using reporter or selectable markers such as fluorescent proteins or drug resistance.

## 7. Conclusions

Identification and understanding of the underlying DNA methylation changes that occur during the early stages of tumorigenesis and the events that drive metastasis are crucial in establishing the role of epigenetics in cancer. CRISPR-based tools that induce targeted methylation and demethylation will be able to decipher the links between transcriptional regulation and DNA methylation status. It is also hoped that these will pave the way for development of epigenetic-based strategies beneficial for cellular engineering and for therapeutic applications in future.

## Figures and Tables

**Table 1:** Hallmarks of cancer and examples of genes silenced by aberrant methylation.

Hallmark	Gene	Gene Function
Self-sufficiency in growth signals	*RASSF1A*	Regulation of Ras pathway [31]
Evading apoptosis	*Caspase-8*	Initiation of apoptosis [32]
Insensitivity to antigrowth signals	*p16/CDKN2A*	Cyclin-kinase inhibitor [33]
Tissue invasion and metastasis	*VHL (Von Hippel-Lindau)*	Suppression of metastasis [34]
Sustained angiogenesis	*VEGF-2*	Crucial for angiogenesis [35]
Limitless replicative potential	*RB (Retinoblastoma)*	Cell cycle regulation [36]

**Table 2:** Summary of CRISPR-dCas9-based tools for DNA methylation.

dCas9 Tool	Feature
dCas9-DNMT3A	Targeted CpG methylation-altered CTCG looping and local gene expression [85]
dCas9-DNMT3ACD	Targeted CpG methylation of the promoter silences gene expression; high off-target DNA methylation is observed using unspecified sgRNAs [77]
dCas9-DNMT3ACD-DNMT3L	Multimerization of DNMT3A-DNMT3L complexes on the promoter to induce long term hypermethylation and gene silencing [78,86]
dCas9-DNMT3ACD, DNMT3L, KRAB	Triple-engineered transcriptional repressors (ETRs): Using a combination of Cas9-DNMT3A, dCas9-DNMT3L and dCas9-KRAB to promote long-term silencing of endogenous genes [79]
dCas9-SunTag-DNMT3A	SunTag recruits multiple copies of antibody-fused DNMT3A to increase CpG methylation [83]
dCas9-MQ1	In vivo application in mice by zygote microinjection [84]
dCas9-SunTag-DNMT3ACD	Modular SunTag shows reduction of off-target events [87]
dCas9-Split M.SssI	Catalytic domain is split for higher specificity [88]

**Table 3:** Summary of CRISPR-dCas9-based tools for DNA demethylation.

dCas9 Tool	Feature
dCas9-TET1CD	Targeted demethylation of the *BRCA* promoter activates gene expression [91]
dCas9-TET1CD, MS2-TET1CD	Modified sgRNA (sgRNA2.0) were constructed using bacteriophage MS2 RNA elements [92]
dCas-TET1CD	Demethylation of CGG repeats induced an active chromatin conformation [94]
dCas9-SunTag-TET1CD	The linker length of original SunTag was changed to 22 amino acids, improving targeted demethylation efficiency [93]
Gal4-ROS1CD	Direct removal of 5mC is induced by ROS1CD glycosylase, without hydroxymethylation [95]
dCas9-R2	A short RNA sequence with stem-loop structure is fused to the sgRNA scaffold and binds DNMT1, inhibiting DNMT1 action to prevent DNA methylation [96]

## Abbreviations

5caC	5-carbocylcytosine
5fC	5-formylcytosine
5hmC	5-hydroxymethylcytosine
5hmU	5-hydroxymethyluracil
5mC	5-methylcytosine
6mA	6-methyladenine
AID	activation-induced deaminase
Ala	alanine
APOBEC	apolipoprotein B mRNA-editing enzyme complex
Asp	aspartic acid
ATP	adenosine triphosphate
BACH2	broad complex-tramtrack-bric a brac and Cap’n’collar homology 2
BCL2	B cell lymphoma 2
BER	base excision repair
bp	base pair
BRCA1	breast cancer 1
C	cytosine
CH3	methyl group
ChIP	chromatin immunoprecipitation
CpG	cytosine-guanine dinucleotide
CRISPR	clustered regulatory interspaced short palindromic repeats
crRNA	CRISPR RNA
dCas9	deactivated Cas9
DNA	deoxyribonucleic acid
DNMT	DNA methyltransferase
DNMTi	DNA methyltransferase inhibitor
EBF3	early B cell factor 3
FMR1	fragile X mental retardation 1
FoxP3	Forkhead box P3
G	guanine
GATA4	GATA binding protein 4
Gly	glycine
GPR17	G protein-coupled receptor 17
gRNA	guide RNA
HIF-1α	hypoxia-inducible factor 1-alpha
His	histidine
HOXA	homeobox A
HOXD12	homeobox D12
hTERT	human telomerase reverse transcriptase
Igf2/H19	insulin-like growth factor 2/H19
KRAB	Krupple-associated box
MAGE	melanoma antigen gene
MGMT	O-6-methylguanine-DNA methyltransferases
mRNA	messenger RNA
MTase	methyltransferase
MyoD	myoblast determination protein 1
p16/CDKN2A	p16^INK4a^/cyclin-dependent kinase inhibitor 2A
PAM	protospacer adjacent motif
PCR	polymerase chain reaction
RASSF1A	Ras association domain-containing protein 1
RB	retinoblastoma
RNA	ribonucleic acid
RNA-seq	RNA-sequencing
ROS1CD	ROS1 5mC DNA glycosylase
RT-qPCR	quantitative reverse transcription PCR
SAH	S-adenosyl-homocysteine
SAM	S-adenosyl-L-methionine
Ser	serine
sgRNA	single-guide RNA
SMRT	single-molecule real-time
SunTag	SUperNova TAGging
TALE	transcription activator-like effector
TDG	thymine DNA glycosylase
TET	ten-eleven translocation
TIMP2	tissue inhibitor of metalloproteinases 2
tracrRNA	trans-activating CRISPR RNA
UHRF1	ubiquitin-like containing PHD and RING finger domains 1
VEGF-2	vascular endothelial growth factor 2
VHL	Von Hippel-Lindau
WT1	Wilms tumor 1
ZNF	zinc finger
